# Multiple sclerosis in a multi-ethnic population from Northern California: a retrospective analysis, 2010–2016

**DOI:** 10.1186/s12883-020-01749-6

**Published:** 2020-04-30

**Authors:** Robert J. Romanelli, Qiwen Huang, Joseph Lacy, Lobat Hashemi, Alana Wong, Alden Smith

**Affiliations:** 1 Sutter Health, Palo Alto Medical Foundation Research Institute, Center for Health Systems Research, Palo Alto, CA USA; 2 Department of Neurology, Sutter Health, Palo Alto Foundation Medical Group, Palo Alto, CA USA; 3 Sanofi Genzyme, Cambridge, MA USA

**Keywords:** Multiple sclerosis, Electronic health records, Real-world; race-ethnic disparities

## Abstract

**Background:**

Research is needed to examine differences in multiple sclerosis (MS) prevalence by race-ethnicity. The goal of this study was to quantify MS prevalence in a health care system in Northern California and examine differences in prevalence and phenotype by race-ethnicity.

**Methods:**

We conducted a retrospective, observational cohort study of adults (2010–2016). MS prevalence estimates were standardised to distributions of gender and race-ethnicity for the underlying geographic region and stratified by gender and race-ethnicity with age adjustment. We performed a chart review of a racial-ethnic stratified sample of patients to examine disease phenotypes.

**Results:**

1,058,102 patients were identified, of which 3286 had MS. The overall direct-standardised prevalence was 288.0 cases per 100,000 population (95% confidence interval: 276.3–299.8). Age-adjusted prevalence ranged from 677.0 per 100,000 among non-Hispanic black women to 49.7 per 100,000 among non-Hispanic Asian men. Non-Hispanic blacks compared with other groups more often had primary-progressive (10.0% vs. 0.0–4.0%) or progressive-relapsing MS (6.0% vs. 0.0–2.0%).

**Conclusions:**

In this Northern Californian Cohort, between 2010 and 2016 the direct-standardised MS prevalence was estimated at 288.0 per 100,000 population, and increased over time. Non-Hispanic blacks, especially women, were disproportionately affected and had less common, earlier progressive MS phenotypes.

## Background

Multiple sclerosis (MS) has been estimated to affect > 400,000 adults in the United States (US) [1], but more recently this number was estimated at approximately 730,000 [2], nearly twice what was previously calculated. The utility of national estimates of MS, however, is limited, as such estimates appear to differ by geographic region. For example, the prevalence of MS is reported to be twice as high in the northern US compared with the southern US [1], which is consistent with other studies showing higher MS prevalence at colder, more extreme, northerly latitudes [3–5].

The burden of MS has been shown to differ by race and ethnicity, with persons of African descent generally having a lower risk of MS than Caucasians [6]; however, this evidence is mostly based on ecological studies. In a more recent study from Southern California, MS incidence was found to be higher among African Americans than non-Hispanic whites (NHWs) between 2008 and 2010 (10.2 vs. 6.9 cases per 100,000 person-years, respectively), particularly among African American women (14.7 per 100,000 person-years) [7]. Prevalence and incidence of MS consistently have been shown to be lower among Hispanics and Asians relative to Caucasians [6–8].

Racial-ethnic differences in MS underscore the complex interaction between genetic, biological, and environmental factors in the etiology of this disease. More studies are needed to understand differences in MS prevalence between diverse racial-ethnic groups residing in the same geographic region.

## Methods

### Study design and setting

This was a retrospective, observational study conducted with an electronic health record (EHR)–based cohort of patients from Sutter Health, a large, community-based health care delivery system in Northern California, and approved by the Sutter Health Institutional Review Board. Sutter Health provides comprehensive medical services across 22 California counties, representing > 100 rural and urban communities. Annually, Sutter Health has > 11 million ambulatory-care visits and 200,000 hospital discharges. All Sutter Health outpatient clinics (*N* = 130) and acute-care hospitals (*N* = 24) operate under a single EHR (Epic; Verona, WI, USA) and billing system. The catchment area of Sutter Health is diverse, with > 50% of individuals belonging to a racial-ethnic minority group: 26.0% Hispanic of any race, 21.0% Asian, 6.0% African American, and 4.0% other or mixed race.

### Cohort identification

The study cohort was composed of patients ≥18 years of age, with two or more health care encounters of any type at a Sutter Health clinic ≥180 days apart during the study period (January 1, 2010–December 31, 2016). The first health care encounter during the study period was defined as the index date. We further required patients to have one or more office encounters in the 12 to 48 months before the index date to confirm prior health system contact and to collect medical history. We excluded patients with any evidence of neuromyelitis optica (a differential diagnosis of MS) defined on two or more encounter diagnoses ≥180 days apart (International Classification of Disease [ICD]-9: 341.0 or ICD-10: G36.0), or the presence of neuromyelitis optica on a patient’s problem list.

### Outcome measurements

The primary outcome of interest was prevalence of MS, defined as two or more encounter diagnoses (ICD-9: 340; ICD-10: G35) ≥180 days apart during the study period or the presence MS on the patient’s problem list. A detailed description of the operationalisation of MS prevalence is described below.

### Data collection and management

Among eligible patients, we extracted data from the EHR on demographics and clinical characteristics in the 12 months prior to the index date for patients without MS, and 12 months prior to the date of the first MS encounter during the study period for those with MS. Age, gender, race, and ethnicity are self-reported by patients at routine clinical encounters. Race-ethnicity was categorised as 1) Hispanic, of any or unknown race; 2) NHW; 3) non-Hispanic Asian (NHA); 4) non-Hispanic black (NHB); 5) non-Hispanic other (including those who self-reported “other” or those of mixed race); and 6) non-Hispanic unknown (i.e., identification of race was missing). Comorbid clinical conditions, with a focus on autoimmune conditions, were identified by ICD-9/10 codes. A Charlson comorbidity index (CCI) score was calculated for each patient based on established methods [9]. Census block median household income was derived from the 2010 US Census based on patient addresses, and was used as a proxy for socioeconomic status.

The overall (2010–2016) period prevalence of MS and annual prevalence were measured. Prevalence was calculated as the number of MS cases divided by the total number of patients in the observational window. For the overall study period, the total population was defined as all patients meeting cohort eligibility. For each year, the total population was defined as patients with one or more health care office encounters during that year.

A nested chart review was conducted among a random sample of 200 prevalent MS cases in our cohort, stratified by race-ethnicity (*N* = 50, each): Hispanic, NHW, NHA, and NHB. The chart review was performed by a clinical nurse, who extracted information from patients’ medical records on age at onset of MS and MS phenotype: relapse-remitting MS (RRMS), secondary-progressive MS (SPMS), primary-progressive MS (PPMS), or progressive-relapsing MS (PRMS). We attempted to collect data on MS disease severity, as measured using the Expanded Disability Status Scale; however, this was poorly documented in patient charts (data not shown) and analyses were not feasible.

### Statistical analysis

Analyses were performed using SAS v9.4 (SAS Institute; Cary, NC). Continuous variables were summarised as means and standard deviation and categorical variables were summarised as percentages. Prevalence was expressed per 100,000 population.

Direct standardisation of prevalence estimates was calculated based on the gender and racial-ethnic distribution of the underlying population for each of the 20 California counties in which MS cases were identified in this cohort (Alameda, Amador, Contra Costa, El Dorado, Lake, Marin, Merced, Napa, Placer, Sacramento, San Francisco, San Joaquin, San Mateo, Santa Clara, Santa Cruz, Solano, Sonoma, Stanislaus, Sutter, and Yolo) served by Sutter Health, using PROC STDRATE in SAS. Ninety-five percent confidence intervals (CIs) were calculated for direct-standardised prevalence based on the normal distribution. Gender and racial-ethnic distributions by county for each year of the study period were obtained from the US Census Bureau [10]. Trends in standardised annual MS prevalence were examined using linear regression. Period prevalence of MS was calculated for gender and race-ethnicity strata with 95% CIs, after age adjustment using logistic regression.

## Results

### Cohort description

**Fig. 1:**
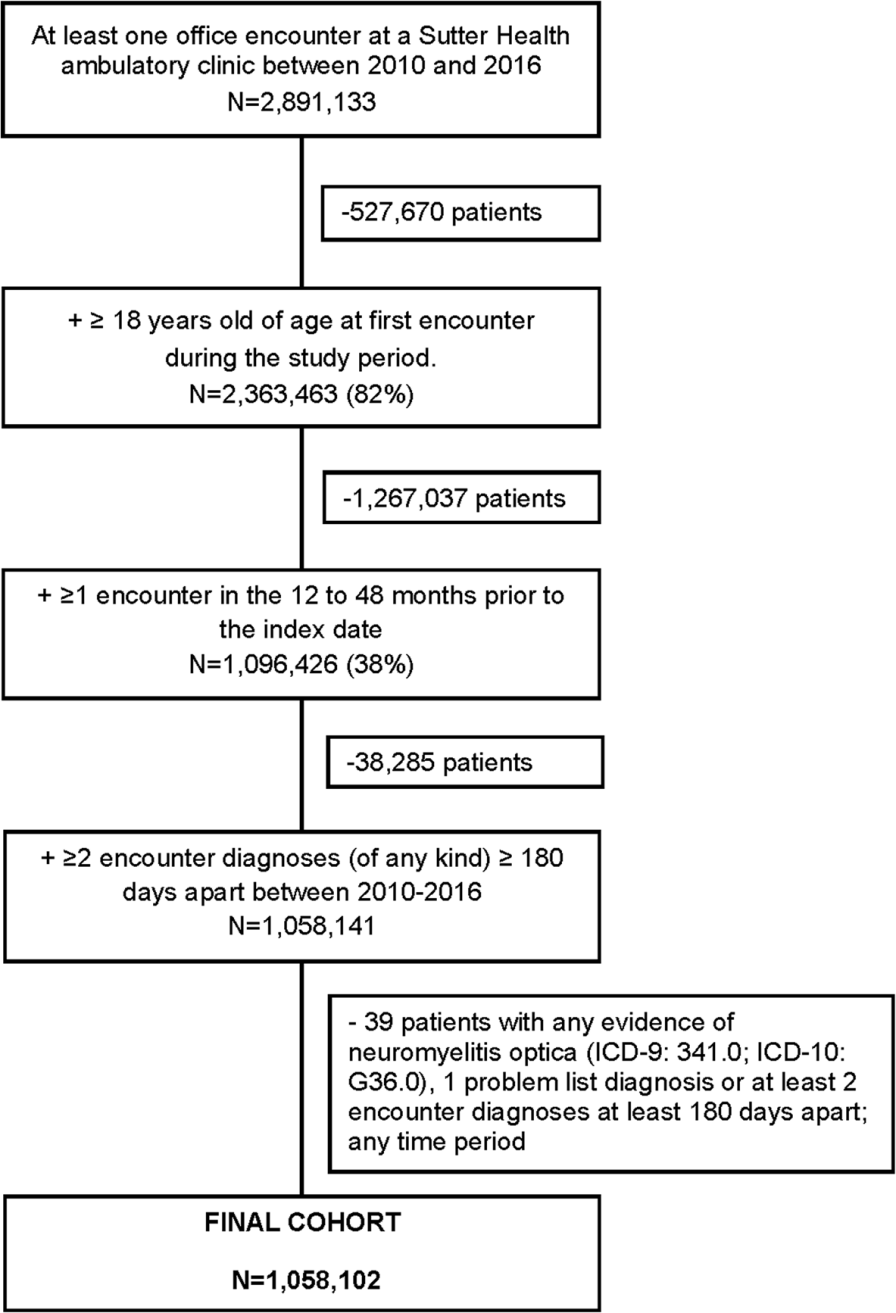
Application of study eligibility criteria. *ICD,* International Classification of Disease

A total of 1,058,102 patients met study eligibility criteria (Fig. 1); 3286 (0.3%) were identified as having MS. MS patients compared with the population without MS were, on average, older (50.6 years vs. 48.0 years) and were more often women (77.7% vs. 60.1%) and NHW (72.0% vs. 55.4%) or NHB (5.2% vs. 3.0%) (Table 1). MS patients were more often ever or current smokers than those without MS (35.4% vs. 25.8%) and more often had thyroid disease (5.7% vs. 4.6%) and depression (9.8% vs. 4.0%), yet they had a CCI score distribution indicating overall less co-morbidity (Table 1).

**Table 1:** Demographics and clinical characteristics by MS status

	No MS *N* = 1,054,816	Prevalent MS *N* = 3286	*P*-value
**Mean age, years ± SD**	48.0 ± 18.4	50.6 ± 12.9	< 0.0001
**Women, n (%)**	633,762 (60.1)	2552 (77.7)	< 0.0001
**Race-ethnicity, n (%)**			< 0.0001
Non-Hispanic white	584,042 (55.4)	2366 (72.0)	
Non-Hispanic black	31,309 (3.0)	170 (5.2)	
Non-Hispanic Asian	136,707 (13.0)	90 (2.7)	
Hispanic	106,661 (10.1)	201 (6.1)	
Non-Hispanic Other	50,642 (4.8)	154 (4.7)	
Non-Hispanic Unknown	145,455 (13.8)	305 (9.3)	
**Median household income, n (%)**			< 0.0001
< $50 K	137,779 (13.1)	479 (14.6)	
$50 K to <$75 K	367,212 (34.8)	1253 (38.1)	
$75 K to <$100 K	304,322 (28.9)	925 (28.1)	
$100 K to $200 K	229,054 (21.7)	581 (17.7)	
> $200 K	2388 (0.2)	5 (0.2)	
Missing	14,061 (1.3)	43 (1.3)	
**Family history of MS, n (%)**	3157 (0.3)	116 (3.5)	< 0.0001
**Smoking history, n (%)**			
Never smoker	660,752 (62.6)	1780 (54.2)	< 0.0001
Ever smoker	194,810 (18.5)	786 (23.9)	
Current smoker	77,208 (7.3)	378 (11.5)	
Unknown	122,046 (11.6)	342 (10.4)	
**CCI score, n (%)**			0.002
0	814,864 (77.3)	2599 (79.1)	
1–2	220,477 (20.9)	655 (19.9)	
3–4	17,695 (1.7)	28 (0.9)	
5–6	1694 (0.2)	4 (0.1)	
> 6	88 (0.01)	0 (0)	
**Mean medications, count ± SD**	0.9 ± 1.4	1.3 ± 1.9	< 0.0001
**Mean BMI, kg/m**^**2**^ **± SD**	27.4 ± 6.4	27.7 ± 6.8	0.07
**Depression, n (%)**	41,785 (4.0)	321 (9.8)	< 0.0001
**Type 1 diabetes, n (%)**	4474 (0.4)	15 (0.5)	0.78
**Thyroid disease, n (%)**	48,972 (4.6)	188 (5.7)	0.003
**Digestive diseases, n (%)**	4843 (0.5)	14 (0.4)	0.78
Ulcerative colitis	1939 (0.2)	5 (0.2)	0.67
Gastroenteritis/colitis	992 (0.1)	0 (0)	0.08
Crohn’s disease	1422 (0.1)	4 (0.1)	0.84
Celiac disease	585 (0.1)	4 (0.1)	0.11
**Rheumatic disease, n (%)**	7263 (0.7)	23 (0.7)	0.94
Lupus	1378 (0.1)	5 (0.2)	0.73
Rheumatoid arthritis	5015 (0.5)	11 (0.3)	0.24
Sjogren’s syndrome	649 (0.1)	6 (0.2)	0.02
Systemic sclerosis	278 (0.03)	1 (0.03)	0.58
Polymyositis	118 (0.01)	0 (0)	1.0
Dermatomyositis	118 (0.01)	0 (0)	1.0

*BMI* body mass index, *CCI* Charlson comorbidity index, *MS* multiple sclerosis, *SD* standard definition

### Main outcomes

**Fig. 2:**
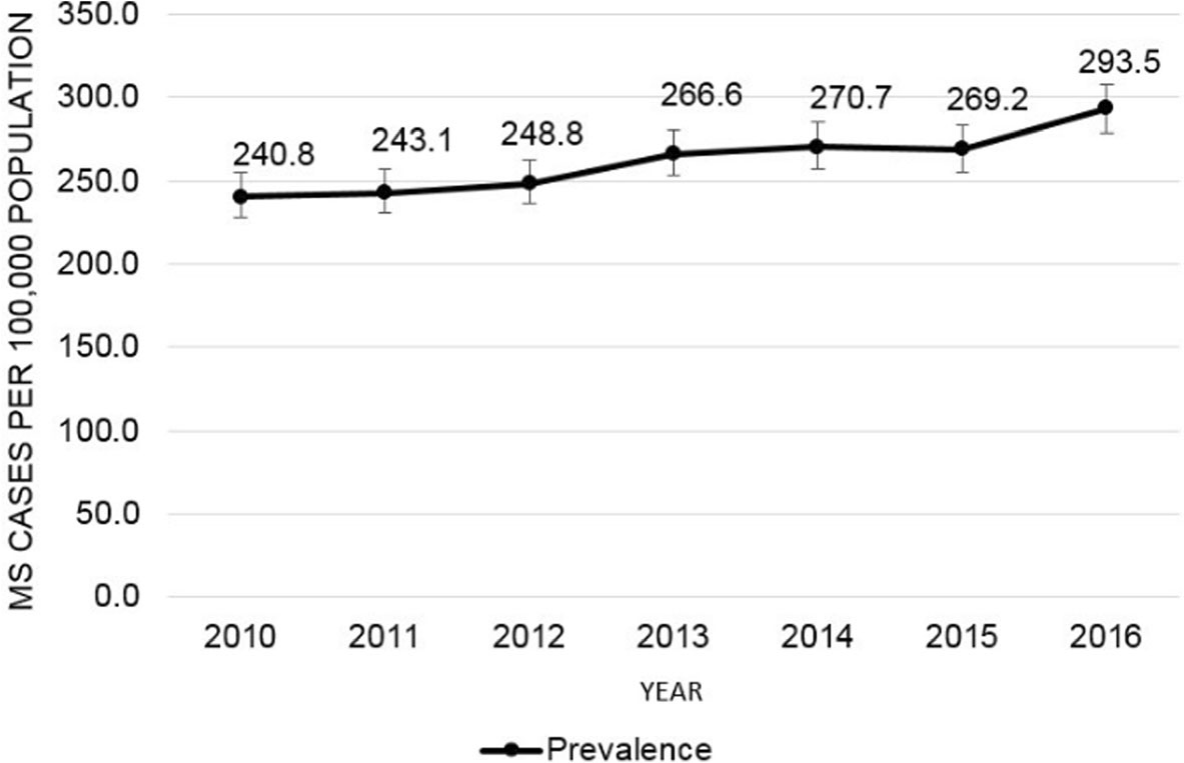
Gender and racial-ethnic standardised annual prevalence of multiple sclerosis. Estimates expressed per 100,000 population. Error bars represent 95% CIs based on the normal distribution. Direct standardised estimates shown for gender (men/women) and racial-ethnic (white, black, Asian, Hispanic, and other) strata based on the distribution in the reference population from the US Census for each year across 20 state counties in Northern California: Alameda, Amador, Contra Costa, El Dorado, Lake, Marin, Merced, Napa, Placer, Sacramento, San Francisco, San Joaquin, San Mateo, Santa Clara, Santa Cruz, Solano, Sonoma, Stanislaus, Sutter, and Yolo. *CIs* confidence intervals, *MS* multiple sclerosis

The direct-standardised period prevalence of MS was 288.0 cases per 100,000 population (95% CI: 276.3–299.8), which increased from 240.8 per 100,000 in 2010 to 293.5 per 100,000 in 2016, representing an average annual increase of approximately 8.8 cases per 100,000 population (*p* < 0.001 for linear trend) (Fig. 2).

**Fig. 3:**
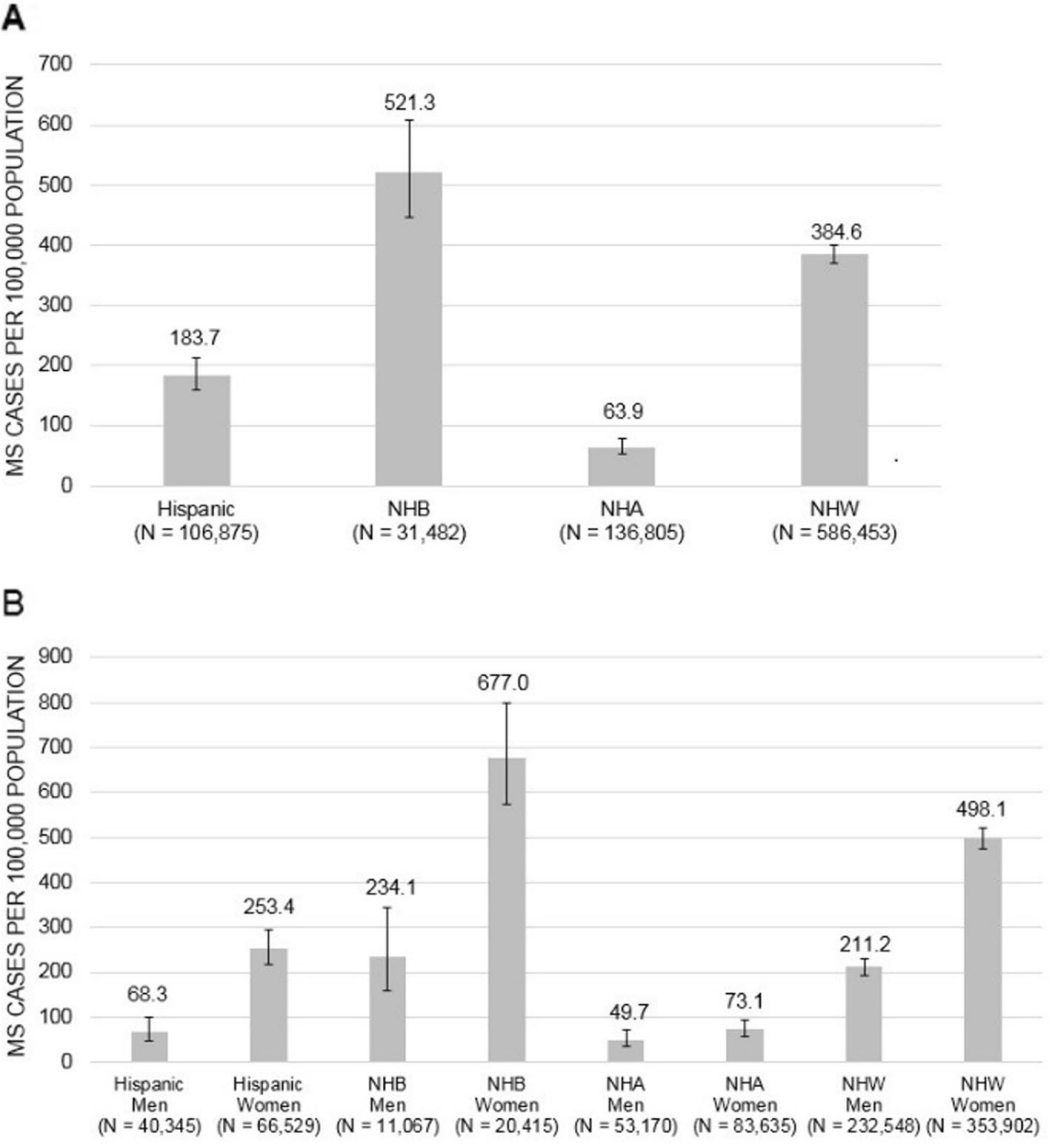
Period prevalence of multiple sclerosis by gender and race-ethnicity, 2010–2016/ Estimates expressed per 100,000 population stratified by race-ethnicity (**a**) and gender/race-ethnicity (**b**), and adjusted for age. Error bars represent 95% CIs based on the normal distribution. *MS* multiple sclerosis, *NHA* non-Hispanic Asian, *NHB* non-Hispanic black, *NHW* non-Hispanic white

The age-adjusted period prevalence of MS was highest among NHBs (521.3 per 100,000) and lowest among NHAs (63.9 per 100,000) (Fig. 3a). Stratified by gender, the age-adjusted period prevalence of MS was highest among NHB women (677.0 per 100,000) and lowest among NHA men (49.7 per 100,000) (Fig. 3b). In women versus men, the prevalence of MS was 3.7-fold higher among Hispanics, 2.9-fold higher among NHBs, and 2.4-fold higher among NHWs (*p* < 0.001 for women vs. men within each racial-ethnic group). MS prevalence was nominally 1.5-fold higher among NHA women than NHA men, but this difference was not statistically significant (*p* = 0.13).

### Chart review

Among a racial-ethnic stratified random sample of 200 prevalent MS cases, mean age at MS onset ranged from 34.6 years in NHWs to 38.5 years in NHBs. RRMS was the most common MS phenotype among NHAs compared with other racial-ethnic groups (62.0% vs. 38.0–52.0%), whereas SPMS was similarly prevalent across groups (16.0–18.0%). PPMS was more common among NHBs than other racial-ethnic groups (10.0% vs. 0.0–4.0%), as was PRMS (6.0% vs. 0.0–2.0%) (Table 2).

**Table 2:** Chart review: Patient demographics and clinical characteristics of patients with MS by race-ethnicity

	Total *N* = 200	Non-Hispanic White *n* = 50	Non-Hispanic Asian *n* = 50	Non-Hispanic Black *n* = 50	Hispanic *n* = 50
**Women, n (%)**	164 (82.0)	41 (82.0)	35 (70.0)	44 (88.0)	44 (88.0)
**Mean age at onset, years ± SD**	36.7 ± 12.0	34.6 ± 9.2	36.1 ± 11.8	38.5 ± 13.5	37.8 ± 12.9
**MS duration, years ± SD**	10.0 ± 10.9	14.8 ± 10.6	7.6 ± 8.3	10.2 ± 11.8	7.6 ± 11.2
**MS phenotype, n (%)**					
Relapse remitting	102 (51.0)	26 (52.0)	31 (62.0)	19 (38.0)	26 (52.0)
Secondary progressive	34 (17.0)	9 (18.0)	8 (16.0)	9 (18.0)	8 (16.0)
Primary progressive	9 (4.5)	2 (4.0)	0 (0)	5 (10.0)	2 (4.0)
Progressive relapsing	4 (2.0)	0 (0)	0 (0)	3 (6.0)	1 (2.0)
Unspecified	46 (23.2)	12 (24.0)	10 (20.0)	14 (28.0)	10 (20.0)
Missing	5 (2.5)	1 (2.0)	1 (2.0)	0 (0)	3 (6.0)

*MS* multiple sclerosis, *SD* standard definition

## Discussion

In a cohort of patients from Northern California, the direct-standardised prevalence of MS was estimated to be 288.0 cases per 100,000 population between 2010 and 2016. Annually during the study period, the prevalence increased. The burden of MS differed by gender and race-ethnicity; NHB women had the highest age-adjusted period prevalence of MS, whereas NHA men had the lowest. Moreover, NHBs had PPMS and PRMS (less common, earlier progressive forms of MS) more often than those in other racial-ethnic groups.

The standardised prevalence of MS in our study was markedly higher than what has been reported previously in the US [1]; yet prior estimates are outdated. Our period estimate is consistent with a more recent statistic from a study by Wallin et al., which, using five large health databases, estimated that MS cumulatively affected approximately 730,000 Americans (309.2 cases per 100,000 population) in 2010 [2]. In our study, the prevalence of MS in 2010 was 240.8 per 100,000, which is consistent, although somewhat lower, than the regional prevalence of 272.7 MS cases per 100,000 (95% CI: 270.1–274.4) in the western US during the same year, as estimated by Wallin et al.

Our findings are also consistent with studies using population-based health administrative data from Canada, which have shown increases in the prevalence of MS over time. In the province of Manitoba, prevalence increased by 49% from 152 cases per 100,000 population in 1998 to 227 per 1000,000 in 2006, representing an average annual increase of 9.4 per 100,000 [11]. In the province of Saskatchewan, prevalence increased by 24% from 254 cases per 100,000 population in 2001 to 314 per 100,000 in 2013, representing an average annual increase of 5.0 per 100,000 [12]. In the province of Ontario, prevalence increased by 69% from 157 cases per 100,000 population in 1996 to 265 per 100,000 in 2013, representing an average annual increase of 6.4 per 100,000 [13]. In our study, we report a similar annual increases in MS prevalence (8.8 per 100,000).

The increase in MS prevalence in our cohort and others may be attributable to changes to diagnostic criteria overall time. However, a decrease in MS-related mortality may also explain such differences. In Ontario, Canada, mortality among those with MS decreased by 33% in the 18-year period between 1996 and 2013 [13]. Population-based studies from both Sweden and Demark have also shown decreasing trends in all-cause mortality among MS patients over several decades (1968 to 2012 and 1950 to 1999, respectively) [14, 15]. However, in the US, at least one study has shown increasing trends in MS-related mortality among both NHWs and NHBs between 1999 and 2015 [16]. Regional, population-based studies are needed on trends in all-cause and MS-related mortality in the US.

To our knowledge, our study is the first to report a higher age-adjusted prevalence of MS in NHBs. These results are consistent with findings from a study conducted in a health care delivery system in Southern California [7]. Prior studies showing lower estimates of MS in individuals of African descent were mostly from ecological studies [6], which can lead to spurious conclusions due to comparisons at the population level rather than the patient level.

The prevalence of MS was consistently higher among women than men in most racial-ethnic groups in our study. The magnitude of gender differences in the prevalence of MS was largest for Hispanics, with Hispanic women having nearly a 4-fold higher prevalence of MS than Hispanic men. NHAs, who had the lowest prevalence of MS, showed no statistically significant differences by gender. Lower estimates of MS among Asians relative to Caucasians have been described elsewhere [6–8]. More studies are needed to understand the genetic and biological factors, and their interactions, that influence different levels of risk of MS within individual racial-ethnic groups.

Approximately 85% of individuals with MS present with RRMS, which often progresses to SPMS, and 10 and 5% of individuals with MS present at disease onset with PPMS and PRMS, respectively [17]. In our chart review, the most common phenotypes of MS were RRMS and SPMS, which together composed 68% of all reviewed cases in our racial-ethnic stratified sample. PPMS and PRMS phenotypes were prevalent in 4.5 and 2.0% of patients, respectively. Approximately 23.2% of patients had an MS phenotype documented as “unspecified” and 2.5% had no MS phenotype documented. The overall lower prevalence of MS phenotypes in our study was likely due to oversampling of racial-ethnic minority groups in the nested chart review, higher variance due to the small sample size (*n* = 200), and a large proportion of patients for whom an MS phenotype was unspecified. However, the relative magnitudes of prevalence of MS phenotypes were consistent with the current literature [17].

Across racial-ethnic groups in our cohort, RRMS was most common among NHAs, whereas rarer, earlier progressive forms of MS, such as PPMS and PRMS, were more common among NHBs. In a retrospective study of African American and white individuals with MS in the US, PPMS (6.8% vs. 4.6%) and, to a lesser degree, PRMS (1.8% vs. 1.5%) were more common in African Americans than whites, respectively, as was SPMS (27.0% vs. 21.6%) [18].

Overall, our study points to potential disparities in MS prevalence and phenotypes across racial-ethnic groups. NHBs are disproportionally affected by MS, with earlier and more progressive phenotypes. Notably, NHBs appear to present with MS at an older age (38.5 years) than other groups (Hispanics, NHA, and NHWs = 37.8, 36.1, and 34.6 years, respectively). However, from our data we cannot determine whether older age at presentation is part of the pathophysiology of disease in NHBs or whether these patients are being diagnosed later. More research is needed to understand the underlying etiology of different MS phenotypes, as well as disease onset and progression in diverse racial-ethnic populations.

Our study has several limitations. The retrospective, observational nature of this study limits causal inferences. Due to the attributes of the study database, we were unable to accurately estimate incidence of MS cases. The chart review was a descriptive analysis and not necessarily powered to detect statistically significant between-group differences. Lastly, we used diagnosis codes to identify MS cases, rather than clinical criteria such as magnetic resonance imaging (MRI) results, which were necessary given that the cohort was derived from an EHR database. To improve the accuracy of identifying MS cases in the database, we required patients to have two or more MS diagnoses ≥180 days apart to mitigate capturing false positives. For example, we attempted to minimise the inclusion of patients who had MS documented in their chart for an encounter at which MS was being assessed, but was not ultimately confirmed as a diagnosis. In our chart review of 200 randomly selected patients, all had confirmed MS. This yielded a positive predictive value of 100%. However, we did not directly assess those classified as not having MS. Thus, we cannot know the false-negative rate (1-sensitivity) of our definition (i.e., proportion of individuals with MS in the population who were not classified as such). The exclusion of these cases would lead to an underestimation of the true prevalence of MS in the population; however, we would not expect misclassification to be different by racial-ethnic group. Our method of calculating prevalence required all patients to have at least two encounters with the healthcare system during the study period (2010–2016), which could impact generalisability of findings given that individuals who do not use the healthcare system are not included. Overall, this is a limitation of a healthcare system-derived cohort.

Despite these limitations, our study has several strengths. We used a large EHR database with comprehensive health care information on > 1 million patients to quantify MS prevalence. Furthermore, gender and race-ethnicity are self-reported in this setting, which strengthens comparisons across gender-racial-ethnic stratified groups. We also standardised period and annual prevalence to the underlying gender and racial-ethnic distributions within each county for better generalisability to the greater Northern California region. Lastly, we were able to perform a review of medical charts on a random sample of patients in our cohort to identify information on MS phenotypes, which cannot be derived from diagnosis codes.

## Conclusions

In a cohort of adults from Northern California, the standardised MS prevalence between 2010 and 2016 was estimated at 288.0 per 100,000 population and increased over time. NHB individuals, especially women, were disproportionately affected by MS and had less common, earlier progressive MS phenotypes.

## Data Availability

Researchers interested in accessing the data from this study are welcome to contact the study investigators. In some circumstances, de-identified aggregated or patient-level data can be supplied to external investigators after appropriate legal, privacy, and Institutional Review Board review and approval.

## Abbreviations

BMI: body mass index
CCI: Charlson comorbidity index
CI: confidence interval
EHR: electronic health record
ICD: International Classification of Diseases
MRI: magnetic resonance imaging
MS: multiple sclerosis
NHA: non-Hispanic Asian
NHB: non-Hispanic black
NHW: non-Hispanic white
PPMS: primary-progressive MS
PRMS: progressive-relapsing MS
RRMS: relapse-remitting MS
SD: standard deviation
SPMS: secondary-progressive MS
US: United States
